# *Ampelopsis japonica* enhances the effect of radiotherapy in non-small cell lung cancer

**DOI:** 10.1007/s00066-024-02322-7

**Published:** 2024-12-04

**Authors:** Zhaohua Liu, Peixia Cui, Qian Wu, Xiao Ji

**Affiliations:** 1 Department of Radiotherapy, Shanxi Province Cancer Hospital/Shanxi Hospital Affiliated to Cancer Hospital, Chinese Academy of Medical Sciences/Cancer Hospital Affiliated to Shanxi Medical University, 030013 Taiyuan, Shanxi China; 2 Orthopedics Department, Affiliated Hospital of Shanxi University of Chinese Medicine/Shanxi Hospital, Xiyuan Hospital of the China Academy of Chinese Medical Sciences, 030024 Taiyuan, Shanxi China; 3 Traditional Chinese Medicine Department, Shanxi Province Cancer Hospital/Shanxi Hospital Affiliated to Cancer Hospital, Chinese Academy of Medical Sciences/Cancer Hospital Affiliated to Shanxi Medical University, 030013 Taiyuan, Shanxi China

**Keywords:** *Tumor growth*, Apoptosis, Radiation therapy, PI3K-Akt signaling pathway, Medicine, Chinese traditional

## Abstract

**Background:**

Radiotherapy is widely used in the clinical treatment of non-small cell lung cancer (NSCLC); however, its effectiveness often proves unsatisfactory. *Ampelopsis japonica* (AJ) is a traditional Chinese herb with anti-inflammatory and anticancer activities. However, whether AJ could enhance the effect of radiotherapy in NSCLC needs to be further explored.

**Methods:**

In vivo, BALB/c nude mice were used for a xenograft tumor model to explore whether AJ could enhance the effect of radiation therapy (RT) in NSCLC. In vitro, human NSCLC cell lines HCC827 and H1299 were used to explore the effect of AJ on the cell proliferation and apoptosis of RT-treated NSCLC. Moreover, bioinformatic analysis was performed to analyze the signaling pathways regulated by AJ.

**Results:**

*Ampelopsis japonica* enhanced the inhibitory effect of RT on NSCLC tumor growth in vivo. Simultaneously, AJ further enhanced the inhibitory effect of RT on NSCLC proliferation and the promoting effect of RT on NSCLC apoptosis. Bioinformatic analysis showed that AJ regulated the PI3K-Akt signaling pathway. We confirmed that AJ decreased the protein levels of the PI3K-Akt signaling pathway. Furthermore, the combination of AJ and RT suppressed activation of the PI3K-Akt signaling pathway.

**Conclusion:**

*Ampelopsis japonica* augmented the inhibitory impact of RT on NSCLC cell proliferation and tumor growth by suppressing the PI3K-Akt signaling pathway.

## Highlights

- 1. *Ampelopsis japonica* suppresses cell survival and proliferation of NSCLC in vitro.
- 2. *Ampelopsis japonica* promotes cell apoptosis of NSCLC in vitro.
- 3. *Ampelopsis japonica* enhances the effect of RT in NSCLC cells by regulating the PI3K-Akt signaling pathway.
- 4. *Ampelopsis japonica* suppresses tumor growth of NSCLC in a xenograft model mice.

## Introduction

Lung cancer remains the leading cause of cancer-related death worldwide, with non-small cell lung cancer (NSCLC) being the dominant form of primary lung cancer [1, 2]. In recent years, great progress has been made in the field of lung cancer treatment, including surgical resection, targeted therapy, chemotherapy, immunotherapy, and radiotherapy [3–5]. However, the overall recovery and survival rates of NSCLC are still not ideal [6, 7]. Therefore, it is imperative to promote research into combined treatment with drugs and radiotherapy in order to expand the scope of clinical benefits and further improve the overall curative effect in NSCLC.

Traditional herbs have provided treatments for a variety of diseases, including cardiovascular diseases, diabetic nephropathy, acute infectious diseases, and cancer [8–11]. Moreover, there is growing evidence that certain herbs can improve the efficacy of cancer treatment and reduce side effects [12, 13]. *Dioscorea nipponica* Makino decreases proliferation and migration of lung cancer cells in vitro [14]. *Scutellaria baicalensis* Georgi plays an anticancer role in lung cancer by inducing apoptosis, organizing the cell cycle, inhibiting proliferation, and blocking invasion [15]. Cucurbitacin B inhibits NSCLC metastasis by reversing epithelial–mesenchymal transition (EMT) progression in gefitinib-resistant cells [16]. *Ampelopsis japonica* (AJ) is a traditional Chinese herb used for the treatment of inflammation, empyrosis, and pain [17–19]. Research of Yuan et al. indicated that AJ inhibited the proliferation and incursion of colorectal cancer by regulating the expression of lncRNA ZFPM2-AS1/miR-515-5p [20]. Nevertheless, whether AJ affects the progression of NSCLC is still unknown.

The purpose of this article is to investigate the effect of AJ extract on tumor growth in vivo and cell proliferation in vitro in NSCLC and to explore and elucidate the possible signaling pathways.

## Materials and methods

### Preparation of AJ extract

Dry AJ powder (Shanghai Yuanye Bio-Technology Co., Ltd., China) was extracted with 70% ethanol using heat reflux extraction three times (for 3 h, 2 h, and 1 h, respectively), followed by decompressing concentration to obtain the concentrated extract. The extract was then filtered, freeze-dried, and stored at −4 °C. The freeze-dried powder was dissolved in 10% dimethyl sulfoxide (DMSO, Beyotime, Shanghai, China) and then filtered through a 0.22-μm syringe filter to form a reserve solution.

### Cell culture and treatment

Human NSCLC cell lines HCC827 and H1299 were purchased from Procell (Wuhan, China) and cultured in Roswell Park Memorial Institute (RPMI)-1640 containing 10% fetal bovine serum (FBS, Thermo Fisher Scientific, MA, USA) and 1% penicillin-streptomycin solution (Procell) under 5% CO_2_ at 37 °C. NSCLC cells were treated with AJ at different doses (0–400 μg/mL) for 24 h to detect the toxicity of AJ in NSCLC cells. Cells were then divided into four groups: control: untreated NSCLC cells; AJ: NSCLC cells treated with AJ at 25 μg/mL for 24 h; RT: NSCLC cells treated with RT at 8 Gy for 24 h; RT + AJ: NSCLC cells treated with AJ at 25 μg/mL and RT at 8 Gy for 24 h.

### Cell Counting Kit-8 (CCK-8) assay

After treatment with different concentrations of AJ or RT, NSCLC cells were seeded into 96-well plates at a density of 5 × 10^3^ cells/well and incubated with 5% CO_2_ at 37 °C. After incubation for 24 h or 48 h, 10 μL CCK‑8 reagent (C0037, Beyotime, China) was added to each well and incubated for 1 h at 37 °C. The optical density (OD) value at 450 nm was detected with a microplate reader (BioTek, VT, USA). The cell survival rate was calculated as $\text{Cell}\,\text{survival}\,\left(\mathrm{{\%}}\right)=\frac{\text{ODtreatment}\,\text{group-ODblank}}{\text{ODcontrol}\,\text{group-ODblank}}\times 100{\%}$.

### Colon formation assay

After treatment with AJ or RT, NSCLC cells in the logarithmic growth phase were digested with 0.25% trypsin, resuspended in complete medium, and counted. Subsequently, cells were seeded into 6‑well plates at a density of 1000 cells/well and cultured for 14 days. The medium was changed every 3 days during culture. After cloning, the cells were washed once with phosphate-buffered saline (PBS, Beyotime) and fixed with 1 mL 4% paraformaldehyde for 30 min. After washing once with PBS, the cells were stained with 1 mL crystal violet for 15 min. After washing with PBS several times, the cells were dried and then photographed with a camera. The colony numbers were counted by Image J software (National Institutes of Health, MD, USA).

### Apoptosis detection

Apoptosis of NSCLC cells was detected using the Annexin V‑FITC Apoptosis Detection Kit (C1062S, Beyotime). NSCLC cells were digested with 0.25% trypsin and then transferred into centrifuge tubes. After centrifugation at 1000 g for 5 min, cells were resuspended and counted. A total of 5 × 10^4^ cells were centrifugated at 1000 g for 5 min and then resuspended with Annexin V‑Fluorescein Isothiocyanate (FITC) binding buffer. Subsequently, cells were incubated with Annexin V‑FITC and propidium iodide (PI) for 15 min in the dark at 25 °C. All cell events were gated by P1 to exclude dead cells and cells in aggregation. The apoptosis of cells was analyzed using flow cytometry (Becton, Dickinson and Company, NJ, USA).

### Western blot assay

Total protein of NSCLC cells was extracted using radio immunoprecipitation assay (RIPA) lysis buffer (P0013, Beyotime) and the protein concentration was detected using the Bicinchoninic acid (BCA) Protein Assay Kit (P0011, Beyotime). The protein sample was split on sodium dodecyl sulfate (SDS)-polyacrylamide gel electrophoresis (PAGE) gels and then transferred onto Polyvinylidene fluoride (PVDF) membranes. After blocking with 5% bull serum albumin (BSA), membranes were incubated with primary antibodies overnight at 4 °C and secondary antibody (Goat Anti-Rabbit IgG H&L [HRP], 1:2000, ab6721, Abcam, Cambridge, UK) for 2 h at room temperature. The membranes were visualized using the ECL kit (36208ES60, Yeasen, Shanghai, China) and analyzed by Image J software. The primary antibodies were c‑caspase‑3 (1:5000, ET1602-39, HuaBio, Wuhan, China), Bax (1:20000, ET1603-34, HuaBio), Bcl‑2 (1:5000, ET1603-11, HuaBio), p‑PI3K (1:1000, #13857, Cell Signaling Technology, MA, USA), PI3K (1:1000, #4249, Cell Signaling Technology), p‑AKT (1:1000, #9272, Cell Signaling Technology), AKT (1:1000, #9271, Cell Signaling Technology), p‑NOS (1:1000, #2977, Cell Signaling Technology), NOS (1:1000, #9575, Cell Signaling Technology), and GAPDH (1:1000, ab9485, Abcam).

### Xenograft tumor model

BALB/c nude mice (20 ± 2 g) were purchased from Chengdu Dossy Experimental Animals co., Ltd. All mice were housed in Specific Pathogen Free (SPF) conditions with free access to food and water. The protocols were approved by the ethics committee of our hospital (GPTAP001). The mice were injected subcutaneously into the right hindlimb with 1 × 10^6^ HCC827 cells. Thereafter, the mice were divided into five groups when the longest tumor diameter reached 6–8 mm (day 10). Mice received RT at 8 Gy [21, 22] on days 10, 12, and 14 using an RS 2000pro Biological X‑ray Irradiator (Rad Source). Thus, the total radiation dose was 24 Gy. For radiotherapy, the mice were anesthetized by intraperitoneal injection of 4% chloral hydrate solution at 80 mL/kg (bodyweight) and then secured to a cardboard plate with medical tape. A 15 mm thick protective lead plate with holes was placed on the mice. The size of the lead plate hole is slightly larger than the size of the tumor hole, which ensures exposure of the tumor and protection of the animal organs. At the same time, different doses of AJ (low dose: 5 mg/kg; middle dose: 10 mg/kg; high dose: 20 mg/kg) were administered to the mice by oral gavage once a day for 15 days (days 10–24). Tumor size was measured every 5 days using a vernier caliper and calculated as volume (mm^3^) = length × width^2^/2. The mice were anesthetized with chloral hydrate and sacrificed to collect tumors at the 25th day for weighing.

### Bioinformatic analysis

The active components of *Ampelopsis japonica* (AJ) were analyzed in the Traditional Chinese Medicine Systems Pharmacology Database and Analysis Platform (TCMSP) database [23] (https://old.tcmsp-e.com/tcmsp.php). The screening criteria were oral bioavailability (OB) ≥ 30% and drug-likeness (DL) ≥ 0.18. DrugBank database (https://www.drugbank.ca/unearth/advanced/bio_entities) was used to analyze the targets of the active components of AJ. For the search of lung cancer-related genes, enter “lung cancer” on the GeneCards website (https://www.genecards.org/) to search for lung cancer-related genes and download the gene list. A Venn diagram was used to obtain the overlapping genes between lung cancer-related genes and the targets of AJ, and a total of 71 genes were obtained. Gene Ontology (GO) and the Kyoto Encyclopedia of Genes and Genomes (KEGG) enrichment analysis of these 71 genes was performed using R software (R Foundation, Vienna, Austria) to explore the mechanism of AJ in NSCLC.

### Statistical analysis

The results were shown as mean ± standard deviation (SD) of three independent experiments, each preformed in triplicate. The data were analyzed using GraphPad Prism 8.0 (GraphPad Software, CA, USA). The differences among multiple groups of data conforming to a normal distribution were analyzed using one-way ANOVA followed by Tukey’s test; the differences among multiple groups of data that did not conform to a normal distribution were analyzed using the Kruskal–Wallis test followed by Dunn’s test. *P* < 0.05 was considered statistically significant.

## Results

### *Ampelopsis japonica* suppresses cell survival and proliferation of NSCLC in vitro

**Fig. 1:**
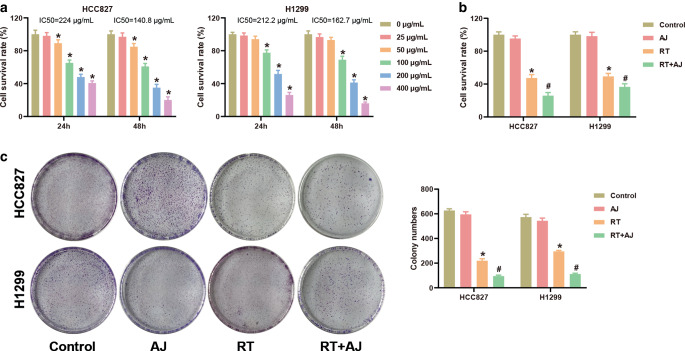
*Ampelopsis japonica* (*AJ*) suppresses cell survival and proliferation of NSCLC in vitro. **a** The cell survival rate of HCC827 and H1299 cells treated by different doses (0 ~ 400 μg/mL) of AJ was detected by CCK‑8 assay. *P < 0.05. **b** The cell survival rate of HCC827 and H1299 cells after treatment with AJ (25 μg/mL) and radiotherapy (*RT*; 8 Gy) was detected by CCK‑8 assay. **c** The proliferation of HCC827 and H1299 cells after treatment with AJ (25 μg/mL) and RT (8 Gy) was detected by colony formation assay. The results are presented as the mean ± standard deviation of three independent experiments, each preformed in triplicate. The differences among groups were analyzed using one-way ANOVA followed by Tukey’s test or by Kruskal–Wallis test followed by Dunn’s test (*P < 0.05 vs. the control group; ^#^*P* < 0.05 vs. the RT group)

The in vitro study was performed to detect the effect of AJ in NSCLC cells. Firstly, we used CCK‑8 to detect the toxic effects of different concentrations of AJ in NSCLC cells. NSCLC cells (HCC827 and H1299) were treated with 1 ~ 400 μg/mL AJ for 24 or 48 h, and the survival rate of NSCLC cells was detected by CCK‑8 assay. As shown in Fig. 1a, the survival rate of NSCLC cells was decreased with an increase in AJ concentration. Among the concentrations, 50 ~ 400 μg/mL AJ showed obvious cytotoxicity to HCC827 cells. The IC50 values of HCC827 cells treated with AJ for 24 h and 48 h were 224 μg/mL and 140.8 μg/mL, respectively. 100 ~ 400 μg/mL AJ showed obvious cytotoxicity to H1229 cells. The IC50 values of H1299 cells treated with AJ for 24 h and 48 h were 212.2 μg/mL and 162.7 μg/mL, respectively. Therefore, AJ at 25 μg/mL was selected for the follow-up study. As Fig. 1b shows, RT markedly decreased survival of NSCLC cells, and AJ administration further enhanced the inhibitory effect of RT on cell survival in NSCLC. Colony formation assay found that RT treatment inhibited proliferation of NSCLC cells, and AJ administration enhanced the effect of RT on cell proliferation of NSCLC (Fig. 1c).

### *Ampelopsis japonica* promotes cell apoptosis of NSCLC in vitro

**Fig. 2:**
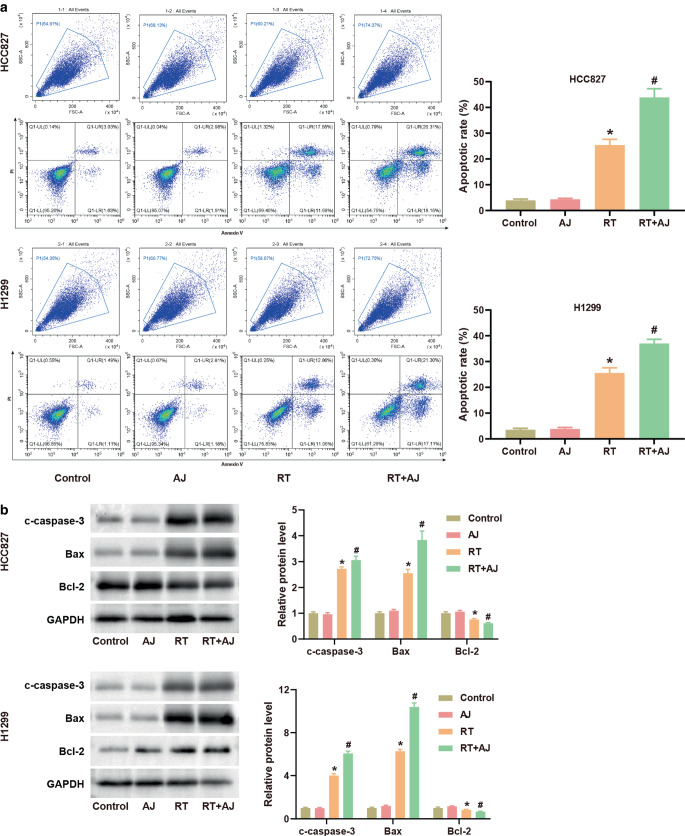
*Ampelopsis japonica* (*AJ*) promotes cell apoptosis of NSCLC in vitro. **a** The apoptotic rate of HCC827 and H1299 cells after treatment with AJ (25 μg/mL) and radiotherapy (RT; 8 Gy) was detected by flow cytometry. **b** The protein expression of c‑caspase‑3, Bax, and Bcl‑2 in HCC827 and H1299 cells after treatment with AJ (25 μg/mL) and RT (8 Gy) was detected by western blot. The results are presented as the mean ± standard deviation of three independent experiments, each preformed in triplicate. The differences among groups were analyzed using one-way ANOVA followed by Tukey’s test or by Kruskal–Wallis test followed by Dunn’s test (*P < 0.05 vs. the control group; ^#^*P* < 0.05 vs. the RT group)

The apoptosis of NSCLC cells was evaluated by flow cytometry. Compared with the control group, the apoptosis rate in the RT group was markedly increased, while apoptosis of the AJ group showed no significant change (Fig. 2a). Notably, the apoptosis rate in the RT + AJ group was further increased compared to the RT group, indicating the strengthening effect of AJ on RT’s apoptosis-promoting effect in NSCLC cells. The expression of the apoptosis-related proteins c‑caspase‑3, Bax, and Bcl‑2 in NSCLC cells was detected by western blot. AJ had no effect on these proteins. In comparison with the control group, RT treatment increased the protein levels of c‑caspase‑3 and Bax, while it decreased the Bcl‑2 level in NSCLC cells (Fig. 2b). Furthermore, the combination of AJ and RT inhibited the protein expression of c‑caspase‑3 and Bax but promoted the protein expression of Bcl‑2 (Fig. 2b).

### Detection and bioinformatic analysis of the active ingredients of *Ampelopsis japonica*

**Fig. 3:**
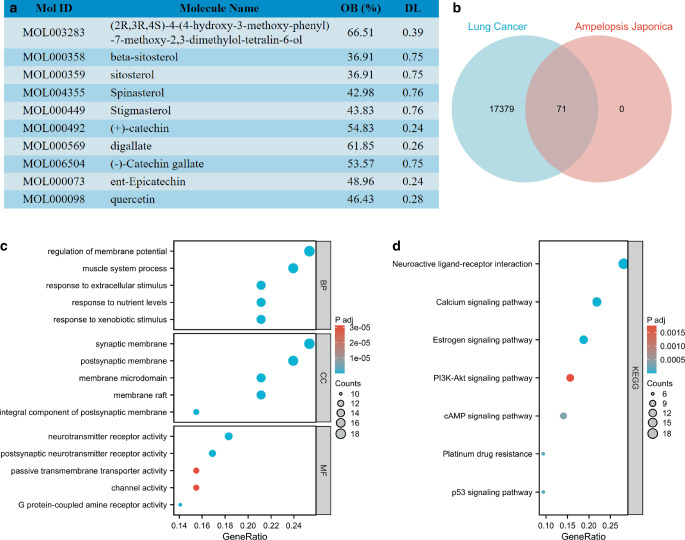
Detection and bioinformatic analysis of the active ingredients of *Ampelopsis japonica* (AJ). **a** The active components of AJ were analyzed using the TCMSP database. The screening criteria were oral bioavailability (*OB*) ≥ 30% and drug-likeness (*DL*) ≥ 0.18. **b** Venn diagram of the lung cancer-related genes and the *Ampelopsis japonica *target genes. The lung cancer-related genes were searched from the GeneCards, and the targets of AJ were analyzed in the DrugBank database. **c** The GO functional enrichment analysis for the selected targets was performed using R software (R Foundation, Vienna, Austria). **d** KEGG pathway enrichment analysis for the selected targets was performed using R software

The active components of AJ were analyzed using the TCMSP database screened according to OB ≥ 30% and DL ≥ 0.18. A total of 10 active components were obtained and listed in Fig. 3a. The DrugBank database was used to analyze the targets of the active components and returned 71 targets. The GeneCards database was used to analyze the therapeutic targets of NSCLC and returned 17,450 targets. The targets obtained from the DrugBank database were intersected with the therapeutic targets obtained from the GeneCards database, resulting in 71 targets for NSCLC (Fig. 3b). GO and KEGG of these targets were analyzed by R. In terms of the biological processes (BP), targets were mainly concentrated into regulation of membrane potential, muscle system process, response to extracellular stimulus, response to nutrient levels, and response to xenobiotic stimulus. In terms of the cell components (CC), targets were mainly concentrated in the synaptic membrane, postsynaptic membrane, membrane microdomain, membrane raft, and in the integral component of the postsynaptic membrane. In terms of the molecular function (MF), most of the targets were rich in neurotransmitter receptor activity, postsynaptic neurotransmitter receptor activity, passive transmembrane transporter activity, channel activity, and G protein-coupled amine receptor activity (Fig. 3c). KEGG enrichment analysis showed that most of the targets were concentrated into neuroactive ligand–receptor interaction, calcium signaling pathway, estrogen signaling pathway, PI3K-Akt signaling pathway, cAMP signaling pathway, platinum drug resistance, and the p53 signaling pathway (Fig. 3d). The PI3K-Akt signaling pathway plays a key role in the development of NSCLC and is closely related to the occurrence and development of tumors. Thus, we then explored the regulatory effect of AJ on the PI3K-Akt signaling pathway.

### *Ampelopsis japonica* enhances the effect of RT in NSCLC cells by regulating the PI3K-Akt signaling pathway

**Fig. 4:**
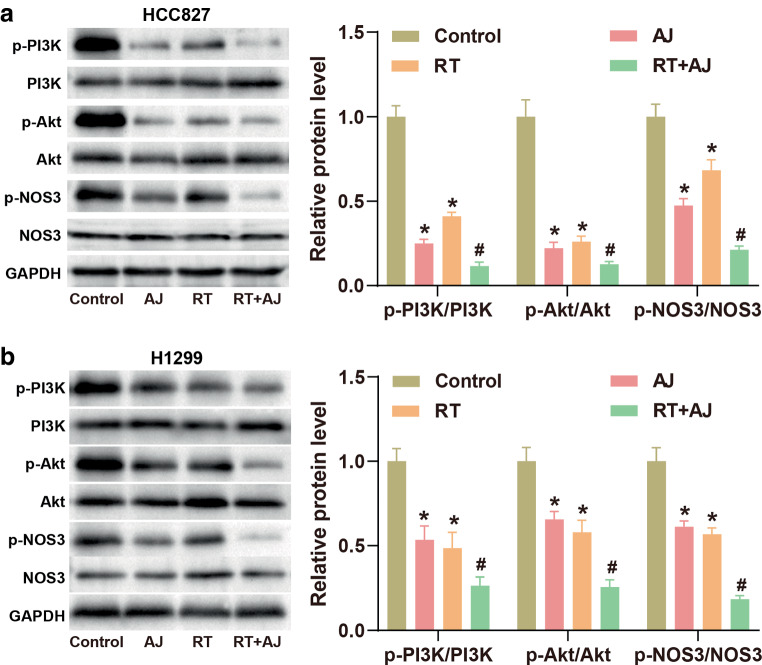
*Ampelopsis japonica* (*AJ*) inhibits the PI3K-Akt signaling pathway. **a**, **b** The protein expression of the PI3K-Akt signaling pathway in NSCLC cells after treatment with AJ (25 μg/mL) and radiotherapy (*RT*; 8 Gy) was detected by western blot. The results are presented as the mean ± standard deviation of three independent experiments, each preformed in triplicate. The differences among groups were analyzed using one-way ANOVA followed by Tukey’s test or by Kruskal–Wallis test followed by Dunn’s test (*P < 0.05 vs. the control group; ^#^*P* < 0.05 vs. RT the group)

In NSCLC, the PI3K-Akt signaling pathway is strongly associated with tumorigenesis and disease progression [24]. To investigate the mechanism of AJ in NSCLC, the protein expression of the PI3K-Akt signaling pathway in NSCLC cells was detected by western blot. As shown in Fig. 4, AJ treatment markedly reduced the expression levels of p‑PI3K/PI3K, p‑Akt/Akt, and p‑NOS3/NOS3 in NSCLC cells compared with the control group, indicating the inhibitory effect of AJ on the PI3K-Akt signaling pathway. Likewise, the levels of p‑PI3K/PI3K, p‑Akt/Akt, and p‑NOS3/NOS3 of the RT group were markedly decreased compared to the control group. Notably, the combination of RT and AJ strengthens the inhibitory effect of AJ or RT on the protein expression of the PI3K-Akt signaling pathway.

### *Ampelopsis japonica* suppresses tumor growth of NSCLC in a xenograft model mice

**Fig. 5:**
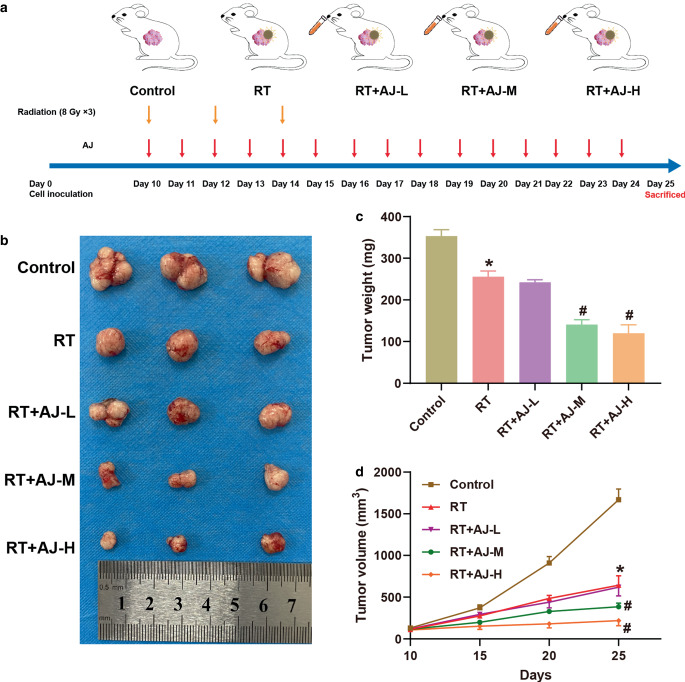
*Ampelopsis japonica* (AJ) suppresses tumor growth of NSCLC in a xenograft model mice. **a** The administration process of the animal experiment. Mice were inoculated subcutaneously with NSCLC cells. Mice of the RT groups were treated with 8 Gy of local RT at day 10, day 12, and day 14. Mice of AJ groups were administered different doses of AJ daily by oral gavage on days 10–24. Tumor tissues were collected at the indicated timepoints for subsequent analysis. **b** Pictures of tumor tissues of each group. **c** Tumor weight of xenograft model mice. **d** Tumor volume of xenograft model mice. The results are presented as the mean ± standard deviation of three independent experiments, each preformed in triplicate. The differences among groups were analyzed using one-way ANOVA followed by Tukey’s test or by Kruskal–Wallis test followed by Dunn’s test (*P < 0.05 vs. the control group; ^#^*P* < 0.05 vs. the RT group)

Nude mice were used to establish a xenograft model to probe the effect of AJ in NSCLC. In the first week following tumor implantation, no significant abnormalities were observed in the spirit and dietary habits of mice in any group. Beginning on the 8th day, as the tumor volume progressively enlarged, the mice in each group began to show adverse effects, including reduced activity, decreased food intake, and delayed responses. At the 10th day, RT and different doses of AJ (AJ‑L, AJ‑M, and AJ-H) were administered to mice. The administration process was shown in Fig. 5a. Following RT and AJ treatments, the activity and mental states of the mice gradually improved, outperforming the control group. The control group exhibited the most rapid growth of transplanted tumors, whereas both the RT and RT + AJ groups showed decelerated tumor growth. The RT + AJ‑H group demonstrated the most pronounced inhibitory effect on tumor growth. Compared with the control group, the tumor weight and volume showed a significant reduction in the RT group (Fig. 5b–d). After AJ administration, the volume and weight of tumor were decreased in a dose–dependent manner (Fig. 5b–d), suggesting that AJ could enhance the inhibitory effect of RT on NSCLC tumor growth.

## Discussion

NSCLC represents the predominant subtype of lung cancer, constituting approximately 85% of all lung cancer cases [25]. Radiation therapy is a commonly employed treatment for patients with NSCLC who are inoperable. Despite advancements in technology, radiation therapy continues to exhibit limitations characterized by suboptimal efficacy and significant toxicity [26]. This paper explores the effect of AJ on radiation therapy for NSCLC, aiming to identify effective complementary and alternative drugs for NSCLC treatment.

In contemporary practice, more and more Chinese herbs are used as adjunctive therapies alongside radiation therapy for cancer patients [27]. Traditional Chinese medicine has been shown to enhance the recovery of normal cellular immunity after radiation therapy, ameliorate the leukopenia and thrombocytopenia induced by radiotherapy, and enhance the radiosensitivity of cancer cells [28]. Chinese herbal injection is an important adjuvant therapy in esophageal cancer radiotherapy, which can significantly improve the clinical symptoms of patients and inhibit the progression of cancer [29]. Curcumin has been shown to reduce the inflammatory toxicity associated with radiation therapy in normal cells and enhance the cytotoxicity of RT in cancer cells by downregulating various anti-apoptotic signaling pathways such as the TGF‑β pathway, PI3K pathway, mTOR pathway, and the NF-κB signaling pathway, thereby enhancing the effect of radiotherapy on the tumor [30]. Studies have shown that AJ extract has a positive effect on the treatment of cancer. Early studies showed that AJ extract can induce apoptosis of promyelocytic leukemia cells [31]. Park, Seung-Man et al. have demonstrated that extracts from AJ exhibit cytotoxic properties on human fibrosarcoma, hepatocellular carcinoma, and breast cancer cells, possibly by activating the activity of macrophages and NK cells [32]. In breast cancer, AJ ethanol extract can inhibit tumor invasion by inhibiting MMP-2/-9 mRNA expression and upregulating TIMP1/2 mRNA expression [33]. In addition, AJ extract inhibited the proliferation, migration, and invasion of colorectal cancer cells by regulating the expression of lncRNA ZFPM2-AS1/miR-515-5p [34]. The study by Su T et al. revealed that AJ ethanol extract enhanced the therapeutic effect of colorectal cancer by inhibiting the β‑catenin signaling pathway [35]. In vitro study revealed that AJ enhanced the effect of radiotherapy in NSCLC cells through mechanisms involving inhibition of cell survival and proliferation coupled with promotion of cell apoptosis of NSCLC. In vivo study further indicated that AJ effectively inhibited tumor growth of NSCLC in xenograft nude mice. These results suggested that AJ may function as an auxiliary agent to potentiate the effect of radiotherapy in NSCLC.

The activation status of the PI3K-Akt signaling pathway correlates closely with both initiation and progression stages in NSCLC pathology [36]. Mechanically, the PI3K-Akt signaling pathway contributes to radiation resistance by accelerating the repair processes of DNA double-strand breaks induced by radiation, enhancing aerobic glycolysis, activating tumor cell proliferation, and diminishing radiation-induced apoptosis [36–38]. Previous studies have shown that targeting inhibition of the PI3K-Akt signaling pathway can enhance the radiosensitivity of glioma cells [39], whereas downregulation of the activity of the PI3K/Akt signaling pathway promotes the radiosensitivity of NSCLC and inhibits tumor development [40]. In our investigation, using GO analysis alongside GSEA, we found enrichment pertaining to active components derived from AJ within key nodes related to the PI3K-Akt signaling pathway. Western blot analysis confirmed substantial suppression mediated by AJ upon expression levels of PI3K-Akt signaling pathway proteins. Moreover, AJ combined with RT further inhibited expression of the PI3K-Akt signaling pathway. The above results demonstrate that AJ could enhance the therapeutic effect of radiation therapy by inhibiting the PI3K-Akt signaling pathway.

## Conclusion

In summary, we demonstrated that AJ is crucial for enhancing the effectiveness of radiation therapy for NSCLC. The mechanism may be to inhibit the PI3K/Akt signaling pathway, thereby enhancing radiation-induced cancer cell apoptosis. AJ may be an attractive candidate for enhancing radiation efficacy and a candidate NSCLC treatment drug. However, the active components of AJ extract are not single, and the mechanism of action of each component needs to be further explored in the future.

## Data Availability

The datasets used and/or analyzed during the current study are available from the corresponding author upon reasonable request.
